# Conditionally Reprogrammed Patient-Derived Nasal Epithelial Cell Cultures for In Vitro Diagnostics and Pharmacological Profiling in Primary Ciliary Dyskinesia

**DOI:** 10.3390/cells15201871

**Published:** 2026-10-15

**Authors:** Ruth M. Urbantat, Anita Balázs, Kathrin Seidel, Marika Drescher, Ricarda Lange, Aditi Löwe, Tihomir Rubil, Julia Duerr, Jobst F. Röhmel, Stephanie Thee, Mirjam Stahl, Marcus A. Mall

**Affiliations:** 1 Department of Pediatric Respiratory Medicine, Immunology and Critical Care Medicine, Charité—Universitätsmedizin Berlin, 13353 Berlin, Germany; ruth-maria.urbantat@charite.de (R.M.U.); anita.balazs@charite.de (A.B.); aditi.loewe@charite.de (A.L.); tihomir.rubil@charite.de (T.R.); julia.duerr@charite.de (J.D.); jobst.roehmel@charite.de (J.F.R.); stephanie.thee@charite.de (S.T.); mirjam.stahl@charite.de (M.S.); 2 German Center for Lung Research (DZL), Associated Partner Site, 13353 Berlin, Germany; 3 Berlin Institute of Health (BIH), 10117 Berlin, Germany; 4 German Center for Child and Adolescent Health (DZKJ), Partner Site Berlin, 13353 Berlin, Germany; 5 Department of Respiratory and Critical Care Medicine, Charité—Universitätsmedizin Berlin, 10117 Berlin, Germany; 6 Cluster of Excellence ImmunoPreCept, Charité—Universitätsmedizin Berlin, 13353 Berlin, Germany

**Keywords:** primary ciliary dyskinesia, conditional reprogramming, air-liquid interface cultures, clenbuterol, β-agonists, diagnostic testing, pharmacological testing, precision medicine, DNAH1

## Abstract

**Highlights:**

**What are the main findings?**

- The conditionally reprogrammed cell (CRC) technology of patient-derived nasal epithelial cells has a high success rate (>90%).
- Air-liquid interface cultures of conditionally reprogrammed nasal epithelial cells recapitulated in vivo ciliary function.
- Primary ciliary dyskinesia (PCD) multiciliated cells exhibit a mutation-dependent response to clenbuterol in vitro.

**What are the implications of the main findings?**

- The CRC technology provides a reliable platform for functional validation of ciliary dysfunction in patient-derived nasal epithelial cells.
- The CRC technology enables genotype-specific therapeutic testing in PCD and is a scalable model for novel drug discovery in PCD.
- The CRC technology is a scalable model for novel drug discovery in PCD.

**Abstract:**

Background: Air-liquid interface (ALI) cultures of patient-derived primary nasal epithelial cells (pNEC) can support diagnostics and in vitro phenotyping of primary ciliary dyskinesia (PCD); however, low cell yield remains a limitation. We aimed to establish and optimize conditionally reprogrammed cell (CRC) technology for pNEC from individuals with PCD and use it as a diagnostic tool for inconclusive cases and for pharmacological testing. Methods: pNEC from 36 individuals (8 healthy controls (HC), 26 PCD, 2 PCD candidates) were expanded using CRC and differentiated at ALI. Ciliary beat frequency (CBF), mucociliary transport velocity (MCTV), and culture morphology were assessed. Further, we assessed the effect of the β-receptor agonist clenbuterol on cilia function in pNEC of HC, PCD (*DNAH11*, *CCDC40*) and PCD candidates harboring biallelic *DNAH1* variants. Results: CRC expansion demonstrated a >90% success rate, yielding high cell counts (mean 9.65 × 10^6^). Differentiation at ALI resulted in pseudostratified ciliated pNEC cultures. CBF was comparable between native and cultured pNEC. MCTV was significantly reduced in PCD vs. HC cultures (*p* < 0.0001). Clenbuterol significantly increased CBF in HC and *DNAH1*, but not in *CCDC40* or *DNAH11* cultures. MCTV was significantly increased in HC by clenbuterol treatment. Conclusions: CRC technology provides a scalable platform for the expansion of pNEC that recapitulate in vivo ciliary function. By enabling genotype-specific pharmacological screening, CRC may serve as a tool for functional diagnostics and precision medicine in PCD.

## 1. Introduction

Primary ciliary dyskinesia (PCD) is a rare, mostly autosomal-recessive monogenic disorder characterized by dysfunctional motile cilia, resulting in impaired mucociliary clearance (MCC) and chronic, progressive, muco-obstructive lung disease from early childhood [1,2,3,4,5,6]. In addition to chronic respiratory disease, PCD may present with laterality defects, chronic rhinosinusitis, otitis media, and subfertility [2,6]. Impaired MCC leads to mucus plugging, persistent bacterial infection, and chronic neutrophilic inflammation, resulting in progressive bronchiectasis and irreversible structural lung damage [2,5]. Mutations in more than 60 genes encoding proteins important for cilia structure and function have been shown to cause PCD [2,7]. The defects result in a spectrum of abnormalities ranging from static cilia to beat amplitude reduction, rotating or hyperkinetic cilia [8]. State-of-the-art diagnostic testing for PCD at specialized centers includes a thorough history, measuring nasal nitric oxide, high-speed video microscopy analysis (HVMA) of nasal epithelial cells to assess ciliary beat frequency (CBF) and ciliary beat pattern (CBP), transmission electron microscopy (TEM) and immunofluorescence staining (IF) to visualize different cilia proteins, and genetic testing (panel sequencing, whole-exome, or whole-genome sequencing) [2,3,6,7,9]. Nevertheless, some cases remain unresolved. To assess and test ciliary function, patient-derived airway cells are needed and can be obtained during bronchoscopy (tracheal or bronchial sampling) or non-invasively by nasal brushings [3,9,10,11].

Respiratory cilia are specialized, motile, hairlike organelles that extend from the apical surface of multiciliated epithelial cells lining the respiratory tract, from the nasal cavity to the terminal bronchioles [2,12]. Ciliary activity can be stimulated by β-adrenergic mimetics that primarily exert their effects through the activation of β2-adrenergic receptors. This signaling cascade triggers an increase in intracellular cyclic adenosine monophosphate (cAMP) and subsequent activation of protein kinase A (PKA), which phosphorylates key components of the ciliary apparatus to enhance CBF and mucociliary transport on airway epithelial cultures and native airways in animal models [13,14,15,16].

Recent advances in PCD diagnostics have led to a higher yield of genetically defined PCD, reducing the proportion of cases with unknown etiology [7,17]. However, in 30–45% of patients with PCD, the genetic defect cannot be identified [2,7]. Therefore, functional and structural studies are important to (i) improve diagnostics, (ii) unravel disease mechanisms, (iii) identify mutation-specific therapeutic targets, and (iv) serve as assays for preclinical testing of novel therapeutic strategies such as emerging messenger RNA (mRNA) replacement therapies [3,18,19,20,21,22]. Freshly isolated nasal epithelial cells obtained from nasal brushings are the gold standard for diagnostic testing [6,11]. Yet, interpretation of the results in fresh brushings can be difficult as ciliary function may be secondarily impaired due to sample handling, infection or inflammation [23,24]. Thus, patient-derived pNEC are cultured at the air-liquid interface (ALI) to eliminate these factors for functional characterization [19,24,25,26]. Major physiological features that are present in vivo, including highly differentiated pseudostratified morphology, composition of relevant fractions of airway epithelial cell types, barrier function, mucus secretion, mucus transport and ciliary beating are retained in ALI cultures [19,25,27,28,29,30]. However, direct culturing of patient-derived cells is limited by the low cell yield of nasal brushings that mainly include differentiated cells and have a limited capacity to proliferate. Diagnostic yield from nasal brushings ranges from 54% to 83% in experienced PCD centers, commonly leading to the need for repetitive nasal brushings and culture for adequate diagnostic testing and functional characterization [10,25,26].

The aim of this study was therefore to use conditional reprogramming of primary nasal cells previously established for cystic fibrosis (CF) to increase cell yield for functional testing and expansion [27,31]. This conditionally reprogramming cell (CRC) technology relies on co-culturing primary epithelial cells with irradiated mouse fibroblast feeder cells and a Rho-associated protein kinase (ROCK) inhibitor (Y-27632), enabling rapid, reversible proliferation while suppressing differentiation and apoptosis [27,28,29,31]. Our primary objective was to establish a scalable ALI culture model that preserves the cell-of-origin function as a diagnostic tool for functional characterization in PCD. Secondarily, we sought to determine if this platform can be used to evaluate the response to pharmacological stimulation with the β_2_-agonist clenbuterol by assessing its effects on CBF and mucociliary transport in cultures from PCD patients with a spectrum of genotypes compared to healthy controls (HC).

## 2. Materials and Methods

### 2.1. Study Population

pNEC were obtained by nasal brushings from 26 patients with genetically confirmed PCD (Table 1 and Table 2), two individuals with suspected PCD, and eight HCs. Demographics of the study population are provided in Table 1. Additional information on the genotype and ultrastructural defect categories is provided in Table 2.

### 2.2. Nasal Brushings

pNEC were obtained from nasal brushings. Prior to sampling, a nasal lavage was performed with sterile 0.9% saline to clear excess mucus and debris. Following the lavage, pNEC were harvested from the medial surface of the inferior turbinate using a sterile celltip cytobrush (Servoprax GmbH, Wesel, Germany). The brush was gently rotated against the mucosal surface to ensure adequate cell yield and immediately transferred to transport medium for downstream analysis or culturing.

### 2.3. Primary Nasal Epithelial Cell Cultures

pNEC obtained by nasal brushings were co-cultured with irradiated ^3T3^fibroblasts in the presence of the RhoA kinase inhibitor Y-27632 in a collagen I coated T75 cell culture flask to 80% confluence as previously described [27,28]. After expansion, cultures at passage 1 were cryopreserved and further passaged. Epithelial cells were seeded at passage 2 or 3 on human placental type IV collagen-coated, 0.4 mm pore size transwell (1.12 or 0.33 cm^2^) inserts (Corning 3460 or 3470, Glendale, NY, USA) at a density of 200,000 cells/cm^2^ and cultured in PneumaCult™ ALI medium (STEMCELL Technologies, Vancouver, BC, Canada) for 21–35 days at ALI.

#### Cell Doubling Time

Cell doubling time was calculated between passage 1 and 2: doubling time = [time in days from passage 1 to passage 2 × (ln2)]/[ln (number of cells at passage 2/number of seeded cells)].

### 2.4. Treatment with Clenbuterol

Highly differentiated pNEC cultures were treated with the β2-adrenoceptor agonist clenbuterol hydrochloride (Sigma-Aldrich C5423, St. Louis, MO, USA). The stock solution was prepared in sterile PBS and diluted in culture medium to a final concentration of 10 µM. Clenbuterol was added basolaterally. For experiments involving acute stimulation, functional assays were performed immediately after adding clenbuterol. For chronic stimulation, cultures were incubated with clenbuterol at 37 °C and 5% CO_2_ in a humidified incubator for 24 or 48 h prior to functional assays. Control cultures received an equal volume of vehicle (PBS) only.

### 2.5. Ciliary Beat Frequency Measurement

CBF was assessed by high-speed video microscopy analysis (HVMA) in nasal cells from fresh brushings and highly differentiated ALI cultures at 120–200 frames per second at 40–100× magnification using an AxioVert.A1 microscope (Zeiss, Jena, Germany) equipped with a monochrome camera (Basler, scA640-120fm or Basler acA1300-um, Ahrensburg, Germany). For HVMA from cultured cells, medium from the basolateral chamber was transferred to the apical chamber and the cell layer was disturbed by scratching off the whole membrane of the insert using a 1000 μL filter tip. Then 500 µL of the medium containing the cell strips was added to a 1.5 mL Eppendorf vial for HVMA. Imaging was performed on a heated stage at 37 °C under a continuous 5% CO_2_ atmosphere. Cell culture inserts (10–20 videos covering the total area), fresh pNEC brushings and post-culture scratched nasal epithelial cells (targeted regions of interest) were analyzed for ciliary function using the Sisson–Ammons video analysis (SAVA) system (Ammons Engineering, Clio, MI, USA) [32]. In addition to CBF analysis, the active area was assessed in pNEC ALI cultures.

### 2.6. Mucociliary Transport Velocity Measurements

To assess mucociliary transport velocity (MCTV), highly differentiated nasal epithelial cultures grown at ALI were first washed with PBS for 30 min to remove mucus. Following the wash, 2 µm fluorescent carboxylate-modified polystyrene beads (FluoSpheres F8825, Thermo Fisher Scientific, Waltham, MA, USA) conjugated to Nile red dye were apically applied at a density of 1500 beads/cm^2^ (~660 beads/µL in PBS) [29]. Cultures were then incubated for at least 48 h without further washing to allow for endogenous mucus accumulation. MCTV was visualized via wide-field fluorescent time-lapse imaging using a Stellaris 8 confocal laser scanning microscope (2.5× air objective, Texas red filter) equipped with an Orca Flash 4.0 V3 sCMOS camera (Hamamatsu Photonics K.K., Hamamatsu, Shizuoka, Japan). Images were captured every second for 60 s and analyzed using the FIJI Trackmate plug-in (ImageJ 1.54p) [33].

### 2.7. Immunofluorescence Staining of Cultures and Cells

#### 2.7.1. Immunofluorescence Staining of Intact Cultures

Culture morphology and cell type composition of the cultures were determined by immunofluorescence (IF) staining as previously described [28]. In brief, cultures were washed with PBS and fixed with 4% paraformaldehyde (28906; Thermo Fisher Scientific, Rockford, IL, USA). Cultures were stained for ciliated cells using an alpha-tubulin antibody (Ab1864, Millipore, Burlington, MA, USA; 1:200), goblet cells using a MUC5AC antibody (mabMUC5AC sc-59951, Santa Cruz, Dallas, TX, USA; 1:200), tight junctions using an Alexa Fluor 555-conjugated anti-Zonula occludens-1 (ZO-1) antibody (MA3-39100-A555, Thermo Fisher Scientific, Waltham, MA, USA; 1:300) and nuclei (Hoechst 33342, Thermo Fisher Scientific, Waltham, MA, USA; 1:5000). Secondary antibodies were used as appropriate (Alexa Fluor 488-conjugated goat anti-rat IgG [SA5-10018, Thermo Fisher Scientific, Waltham, MA, USA] and Alexa Fluor 750-conjugated goat anti-rabbit IgG [A-21039 Thermo Fisher Scientific, Waltham, MA, USA]). Filters were cut out and mounted with ProLong™ Gold anti-fade reagent (P36934, Invitrogen, Thermo Fisher Scientific, Waltham, MA, USA) on a super frost glass slide. Images were acquired with a Stellaris 8 confocal laser scanning microscope (20× water immersion objective) equipped with an Orca Flash 4.0 V3 sCMOS camera (Hamamatsu Photonics K.K., Hamamatsu, Shizuoka, Japan) for wide-field fluorescence imaging.

#### 2.7.2. Immunofluorescence Staining of Cells Post Culture

For immunofluorescence staining, depending on the insert size, 500–1000 µL of Accutase (C-41310, PromoCell distributed through Millipore, St. Louis, MO, USA) was added apically to the cell culture insert and incubated at 37 °C for 30 min. After incubation, cells were resuspended and centrifuged at 1000 rpm for 5 min. Following supernatant aspiration, the pellet was resuspended in 400–800 µL of RPMI-1640 (R8758, Sigma-Aldrich, St. Louis, MO, USA) and aliquoted onto slides (50 µL/slide) [11]. Expression and localization of cilia proteins were assessed using immunofluorescence staining as previously described [11]. Briefly, cells were fixed with 4% paraformaldehyde (28906; Thermo Fisher Scientific, Rockford, IL, USA). After a wash with PBS, the cells were permeabilized (PBS with 0.2% Triton: 100 mL PBS, 200 μL Triton-×100 [T9284, Sigma Aldrich, St. Louis, MO, USA]). After washing with PBS again, the slides were blocked with blocking buffer (PBS with 5% milk powder [T145.2, Carl Roth, Karlsruhe, Germany]) for 1 h at room temperature. Primary antibodies (alpha-tubulin, Ab1864, Millipore, Burlington, MA, USA; DNAH5, HPA037470; GAS8, HPA041311; DNAH1, HPA036806 all Prestige, Sigma Aldrich, St. Louis, MO, USA and RSPH9, NBP1-86750, Novus, Bio-Techne, Minneapolis, MN, USA) were prepared and added in blocking buffer. Cells were incubated overnight at 4 °C in a wet and closed chamber with the primary antibodies. The following day, slides were washed four times with PBS (1× wash down, 1 × 3 min, 2 × 10 min). Secondary antibodies (Alexa Fluor 488-conjugated goat anti-rat IgG [SA5-10018, Thermo Fisher Scientific, Waltham, MA, USA] and Alexa Fluor 594-conjugated goat anti-rabbit IgG [A-11012 Thermo Fisher Scientific, Waltham, MA, USA]) including Hoechst 33342 (1:10.000, Thermo Fisher Scientific, Waltham, MA, USA) were prepared in blocking buffer and added to the slides for 1.5 h at room temperature (protected from light). Afterwards, the antibody solutions were removed and slides were washed four times with PBS (1 × wash down, 1 × 3 min, and 2 × 10 min). Slides were mounted with ProLong™ Gold anti-fade reagent (P36934, Invitrogen, Thermo Fisher Scientific, Waltham, MA, USA) with a coverslip. Images were acquired with a Leica Stellaris 8 confocal laser scanning microscope (20× and 63× water immersion objectives) equipped with a Hamamatsu Orca Flash 4.0 V3 sCMOS camera for wide-field fluorescence imaging.

### 2.8. Statistical Analysis

**Figure 1:**
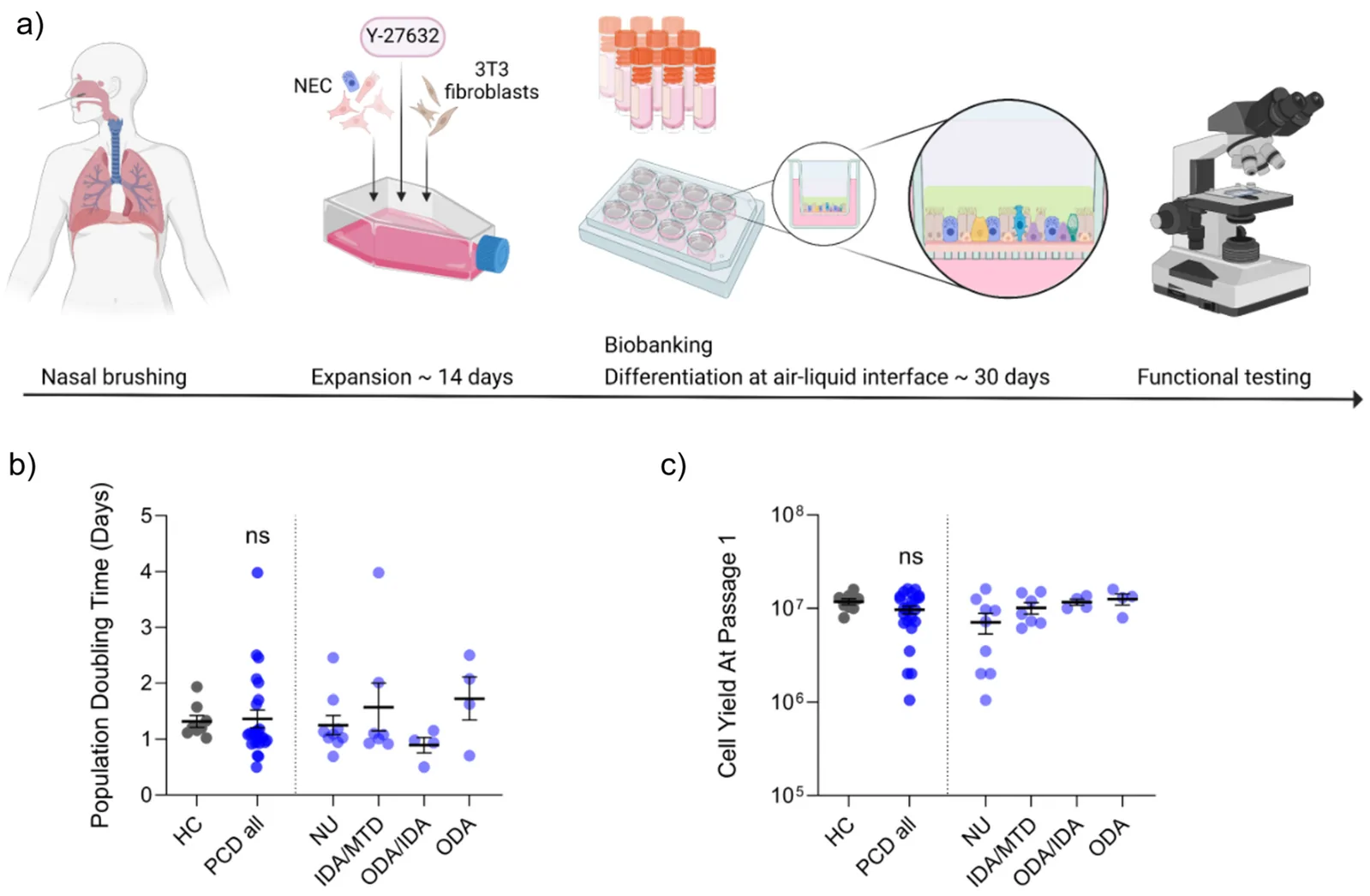
Workflow of conditional reprogramming and culture and yield of highly differentiated nasal epithelial cells. Workflow from nasal brush collection from healthy and PCD donors to nasal epithelial cells expansion, biobanking and/or differentiation at the air-liquid interface (ALI) and functional characterization (**a**). Population doubling time (days) was calculated between passage 1 and 2 (**b**). Cell yield at passage 1 (**c**). Graphs (**b**,**c**) depict mean per donor and show healthy controls (HC) depicted in grey, all PCD (PCD all) depicted in blue as well as PCD donors divided into groups of expected ultrastructural changes (NU = normal ultrastructure, IDA = inner dynein arm, MTD = microtubular disorganization, ODA = outer dynein arm) depicted in transparent blue. HC (*n* = 7) and PCD donors (*n* = 24; NU: *n* = 9; IDA/MTD: *n* = 7; ODA/IDA: *n* = 4; ODA: *n* = 4). For statistical testing data were tested for normality and lognormality distribution and Welch’s *t* test was used to compare healthy controls with PCD donors, ns = not significant. The cartoon (**a**) was created in Biorender.org.

**Figure 2:**
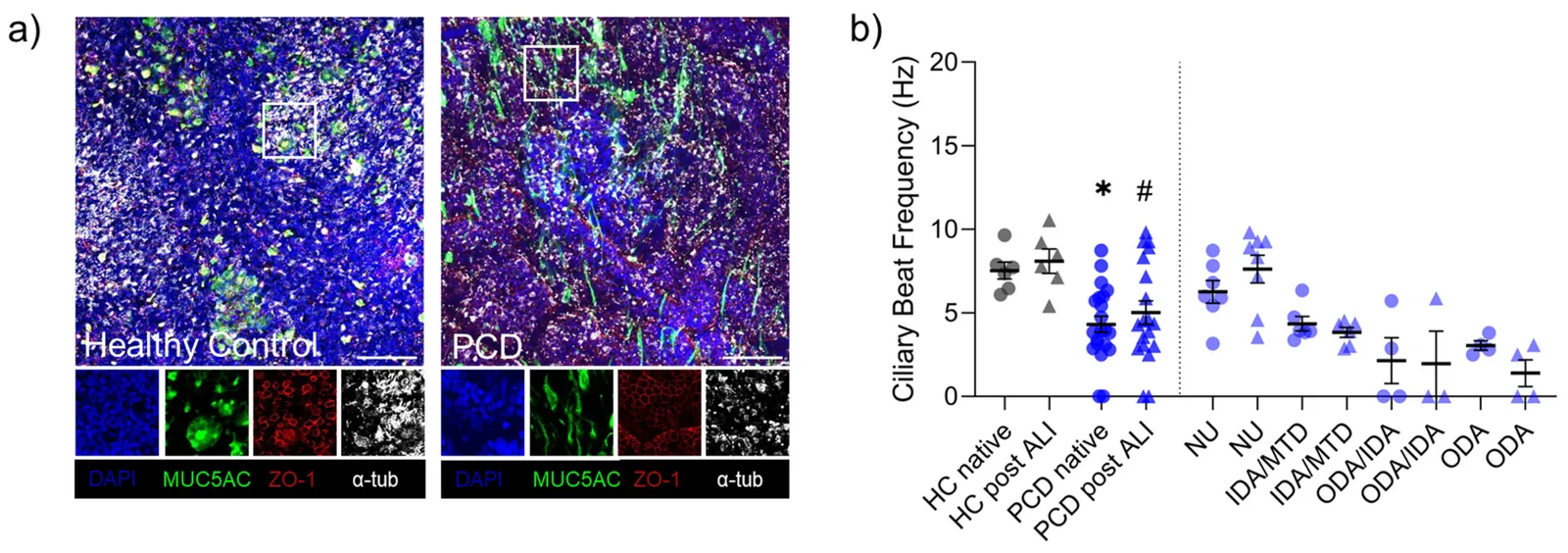
Functional characterization of highly differentiated PCD pNEC ALI cultures. Representative immunofluorescence staining from fixed ALI cultures stained for nuclei (Hoechst = DAPI), goblet cells (*MUC5AC*), tight junctions (*Zonula occludens 1 = ZO-1*) and ciliated cells (*alpha-tubulin*), scale bar 100 µm (**a**). Summary of CBF measurements in native epithelial cells ex vivo obtained from nasal brushings and scratched ALI-differentiated epithelial cells from HC (*n* = 6) in gray and PCD donors in blue (*n* = 22) stratified by ultrastructural defect category depicted in transparent blue (NU: n = 8; IDA/MTD: *n* = 6; ODA/IDA: *n* = 4; ODA: *n* = 4). The dotted line distinguishes between PCD donors and subroup analysis stratified by ultrastructural defect category. For statistical testing, data were tested for normality and lognormality, and differences were evaluated using the Mann–Whitney test. Statistical significance is indicated by: * (*p* ≤ 0.01) for the comparison between native healthy and native PCD samples, and # *p* ≤ 0.05 for the comparison between healthy and PCD samples following ALI differentiation (**b**). NU = normal ultrastructure, IDA = inner dynein arm, MTD = microtubular disorganization, ODA = outer dynein arm.

**Figure 3:**
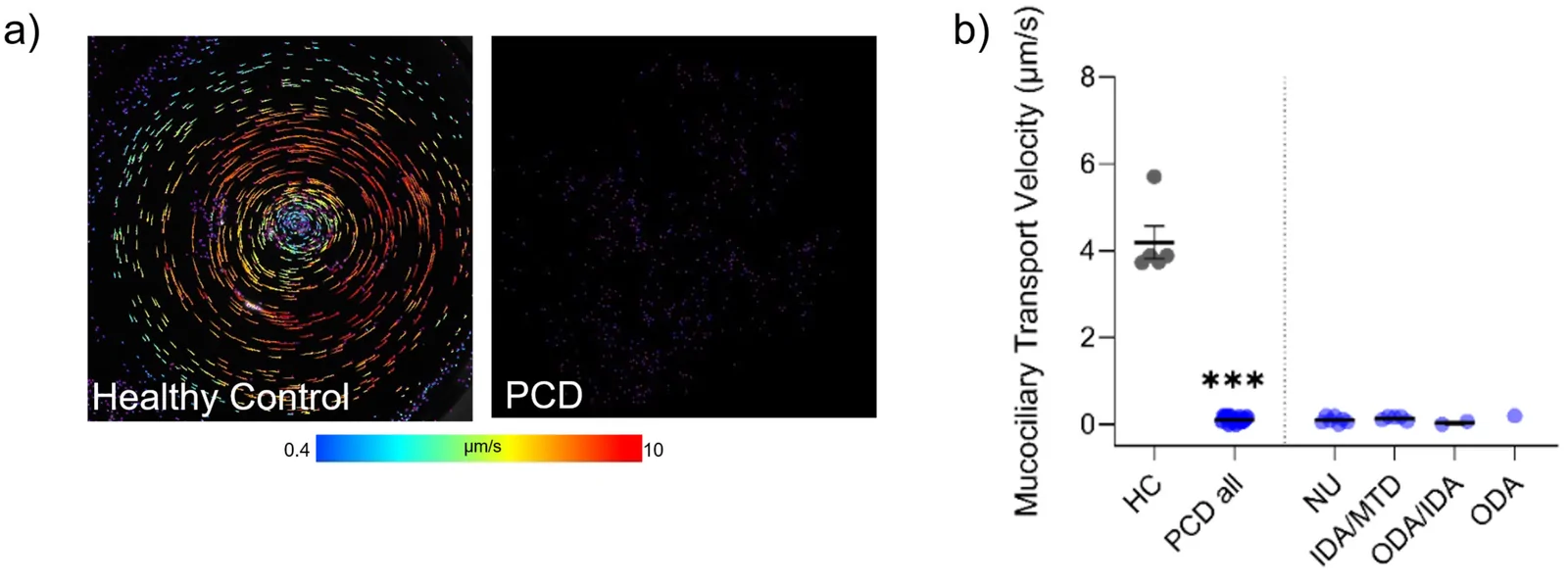
Mucociliary transport velocity (MCTV) is significantly reduced in PCD pNEC ALI cultures compared to healthy controls. Representative bead tracks that visualize MCTV determined by transport rates of fluorescent beads added to the apical surface of differentiated ALI nasal epithelial cultures on cell culture inserts from HC and PCD donors (**a**). Beads are presented in the pseudocolor violet. Summary of measurements depicted as means/donor (**b**); healthy controls (n = 5) depicted in gray, PCD donors (n = 15), depicted in blue and PCD stratified by ultrastructural defect category depicted in transparent blue (NU: *n* = 7; IDA/MTD: *n* = 5; ODA/IDA: *n* = 2; ODA: *n* = 1). The dotted line distinguishes between PCD donors and subroup analysis stratified by ultrastructural defect category.Data are shown as mean per group± SEM; individual data points represent measurements from three to four filters per donor. For statistical testing data were tested for normality and lognormality and the Mann–Whitney test was used for the comparison between cultures from HC and PCD. *** *p* < 0.001. NU = normal ultrastructure, IDA = inner dynein arm, MTD = microtubular disorganization, ODA = outer dynein arm.

**Figure 4:**
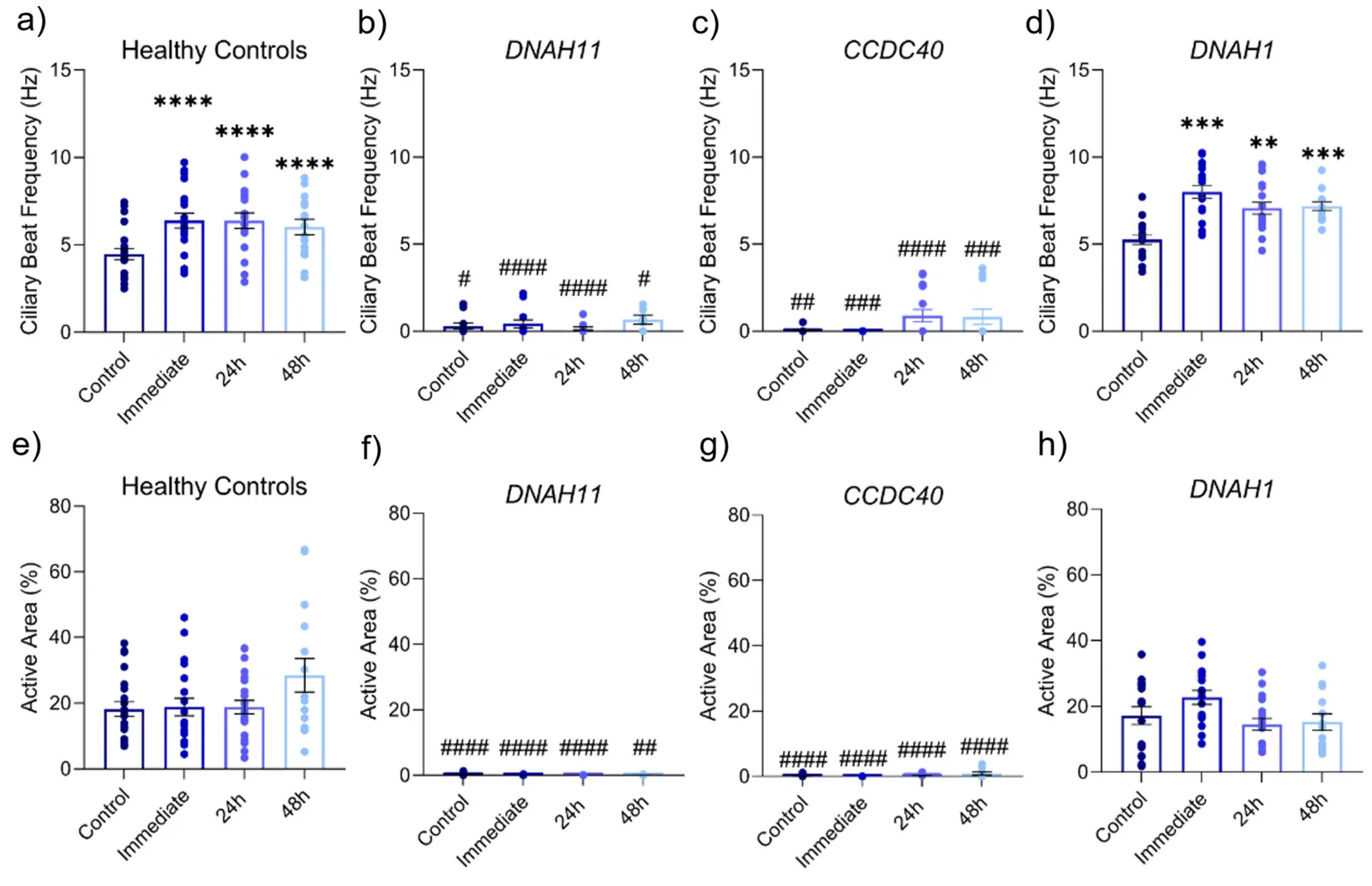
Clenbuterol treatment enhances ciliary beat frequency (CBF) in healthy control and *DNAH1*, but not in *DNAH11* or *CCDC40* ALI cultures. Graphs show the effect of clenbuterol on CBF (**a**–**d**) and the active area, respectively in HC (**a**,**e**), *DNAH11* (**b**,**f**) *CCDC40* (**c**,**g**) and *DNAH1* (**d**,**h**) differentiated ALI cultures on cell culture inserts at different timepoints after basolateral treatment with 10 µM clenbuterol. Measurements are shown as mean CBF ± SEM per filter. HC, *n* = 5; *DNAH11*, *n* = 4; *CCDC40*, *n* = 5; *DNAH1*, *n* = 2. Data for *DNAH*1 were obtained from two to three independent experiments, with 3–4 filters per donor and experiment. For statistical testing within each group, data were tested for normality of distribution before computing mixed-effect analysis with multiple comparisons and Dunnet’s correction. Statistical comparisons were made between treatment and the control group within the same donor (mock treatment), the level of significance is marked by asterisk. For the comparison of HC and different genotypes (*DNAH11*, *CCDC40* and *DNAH1*) at every timepoint, the Kruskal–Wallis test followed by Dunn’s post hoc analysis was performed and is indicated by #; # *p* < 0.05, **/## *p* < 0.01, ***/### *p* < 0.001, ****/#### *p* < 0.0001.

**Figure 5:**
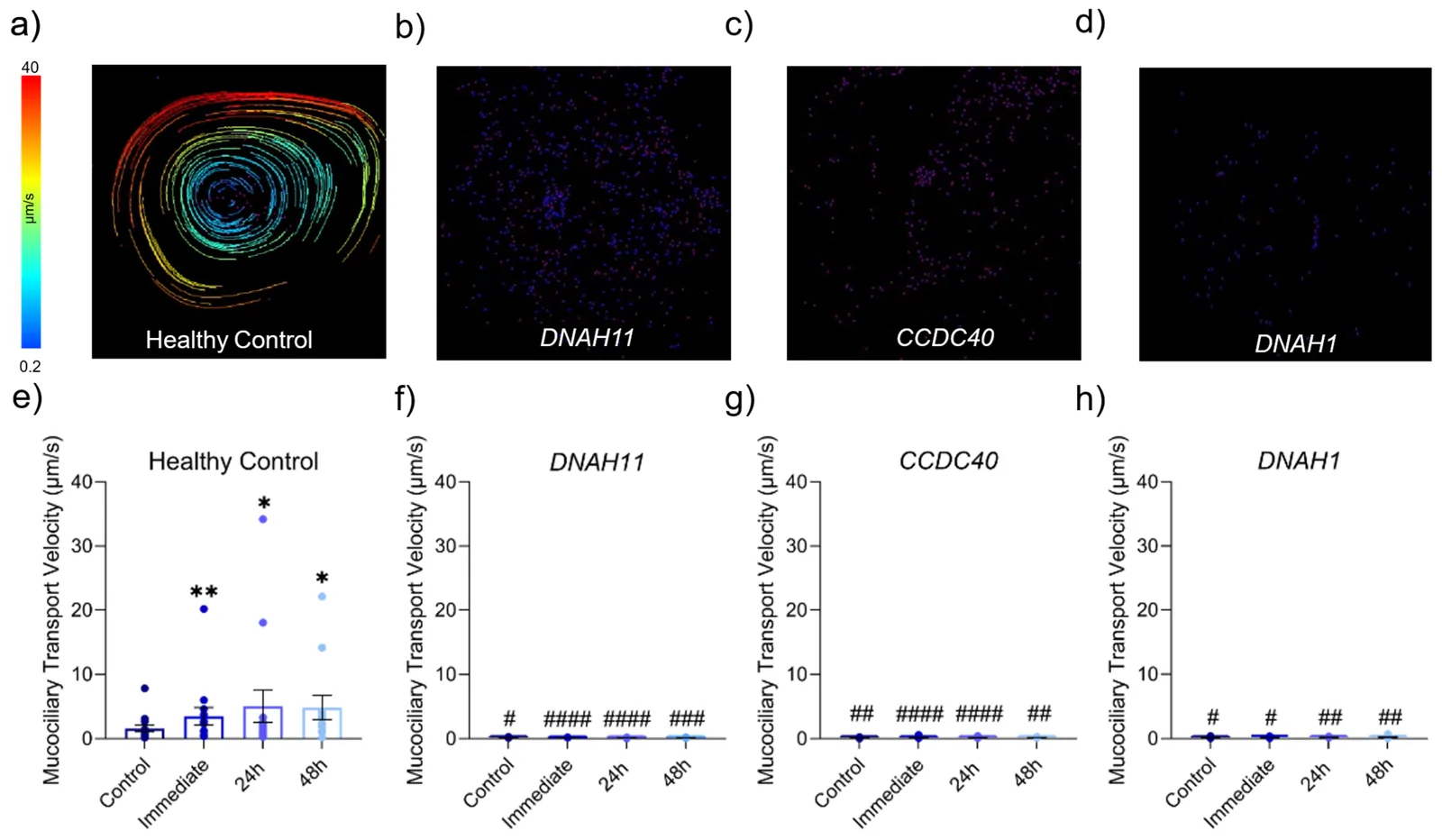
Clenbuterol treatment enhances mucociliary transport velocity (MCTV) in healthy controls, but not in *DNAH11*, *CCDC40* or *DNAH1* ALI cultures. Representative bead tracks that visualize MCTV determined by transport rates of fluorescent beads added to the apical surface of nasal epithelial cultures from healthy controls (**a**), *DNAH11* (**b**), *CCDC40* (**c**) and *DNAH1* (**d**). For better visibility, beads are pseudocolored in purple. Summary of the effect of clenbuterol on MCTV in healthy controls (**e**), *DNAH11* (**f**) *CCDC40* (**g**) and *DNAH1* (**h**) ALI cultures. Summary of measurements depicted as mean per filter ± SEM; healthy controls *n* = 5, *DNAH11* *n* = 4, *CCDC40* *n* = 5, *DNAH1* = 2, with 2 independent repetitions for PCD candidate 2; 3–4 filters per donor per repetition. For statistical testing, data were tested for normality and lognormality, followed by mixed-effects analysis with Holm–Šídák’s multiple comparisons test and Geisser–Greenhouse correction for statistical comparison within a group. Kruskal–Wallis test with Dunn’s multiple comparisons test was used for comparisons between HC and genotypes at corresponding timepoints; * indicates comparison within the group; # indicates comparison to healthy controls and the same treatment group. */# *p* < 0.05, **/## *p* < 0.01, ### *p* < 0.001, #### *p* < 0.0001.

Data were analyzed with Prism 11.0.0 for Windows (GraphPad Software, 11.0.2, Boston, MA, USA). The Anderson–Darling test was used to assess the normality and lognormality of data distribution. Two-group comparisons were performed with two-tailed paired or Welch’s *t* test, Wilcoxon’s test, or the Mann–Whitney test as indicated for comparison between HC and PCD. Multiple-group comparisons were performed using mixed-effects analysis with either Dunnett’s multiple-comparisons test or the Holm–Šidák correction or the Kruskal–Wallis test followed by Dunn’s post hoc comparison as appropriate, and the exact tests are indicated in each figure legend. For studies summarized in Figure 1, Figure 2 and Figure 3, statistical analyses were performed at the donor level. Specifically, when multiple measurements were obtained from cells of the same donor, these measurements were averaged to generate a single mean value per donor, and this donor-level mean was used as the unit of observation for all statistical tests, i.e., each donor contributed one value to the statistical analysis. For studies on the effect of clenbuterol summarized in Figure 4 and Figure 5 donors with variants in DNAH1 were limited to *n* = 2. Therefore, statistical analyses were performed at the filter level. Specifically, each filter was considered an individual unit of observation, and all filters included in the experiments were incorporated into the statistical analyses. *p* < 0.05 was accepted to indicate statistical significance. All data are presented as mean ± SEM.

## 3. Results

### 3.1. Conditional Reprogramming of Nasal Epithelial Cells

#### 3.1.1. Basal Cell Expansion and Differentiation at the Air-Liquid Interface

Nasal brushings were taken from 36 individuals (age range: 3 to 67 years) including eight healthy controls and 26 individuals with PCD (Table 1). After expansion, cultures at passage 1 were further passaged and biobanked (Figure 1a). The success rate was 92.3% (24 of 26) for PCD cultures and 87.5% (7 of 8) for HC.

To assess differences in proliferation potential between nasal cells from PCD donors and healthy controls, the population doubling time was calculated. At passage 2, population doubling times were comparable between pNEC from HC and PCD samples, as well as across different PCD ultrastructural defect categories (Figure 1b; HC: 1.32 vs. PCD 1.37 days). The mean cell yield for HC (11.8 × 10^6^) and PCD donors (9.65 × 10^6^) at passage 1 were similar, with no difference observed regardless of the underlying ultrastructural defect (Figure 1c).

#### 3.1.2. Culture Composition and Functional Testing in Highly Differentiated ALI Cultures

Following conditional reprogramming and ALI culture, highly differentiated pNEC cultures were analyzed by several assays. Culture morphology and cell type composition were determined by immunofluorescence staining. In both HC and PCD cultures, we identified goblet cells (MUC5AC+), ciliated cells (alpha-tubulin+), and a well-defined network of tight junctions (ZO-1+) (Figure 2a).

CBF was measured in native cells from fresh brushings and in dispersed pNEC after culturing at the ALI. In native cells from HC, the mean CBF was 7.25 ± 1.25 Hz (range: 6.09 to 9.64 Hz). CBF in native cells was significantly reduced in PCD compared to HC (HC: 7.25 ± 1.25 Hz vs. PCD: 4.32 ± 2.2 Hz *p* < 0.05) (Figure 2b). In HC, a comparison of ALI-differentiated pNEC and native pNEC revealed comparable CBF values (native: 7.25 ± 1.25 Hz vs. post ALI: 8.09 ± 1.77 Hz; *n* = 6). CBF in ALI-differentiated pNEC was also comparable with the values obtained from initial fresh nasal brushings from PCD individuals (native PCD all 4.32 ± 2.24 Hz, range: 0 to 8.73 Hz; post ALI PCD all: 5.02 ± 3.02 Hz, range: 0 to 9.81 Hz) (Figure 2b). Ciliary motility in PCD pNEC ranged from immotile cilia in both native and ALI-cultured cells from individuals with ODA/IDA or ODA defects to a CBF within the range of healthy controls in cells from individuals with normal ciliary ultrastructure (Figure 2b).

We then determined MCTV by tracking the movement of fluorescent beads via time-lapse imaging on highly differentiated pNEC ALI cultures of healthy controls and PCD donors (Figure 3a,b). In healthy control cultures, beads were transported in a directed manner, whereas PCD cultures did not transport beads (Figure 3a). The mean transport velocity was significantly lower in PCD donors (Healthy: 4.19 µm/s vs. PCD: 0.11 µm/s; *p* < 0.001), independent of the ultrastructural group (NU: 0.10 µm/s; IDA/MTD: 0.14 µm/s; ODA/IDA: 0.03 µm/s; ODA: 0.19 µm/s) (Figure 3b).

### 3.2. Effect of Clenbuterol on Ciliary Beat Frequency and Active Area in Highly Differentiated Air-Liquid Interface Cell Cultures

To determine if CRC cultures can be used for preclinical testing of therapeutic agents that may enhance ciliary beating in PCD, we treated highly differentiated ALI cultures from PCD patients harboring biallelic pathogenic mutations in *CCDC40* and *DNAH11* with the β-agonist clenbuterol and assessed the effects on ciliary function compared to healthy controls (Table A1). These genotypes were selected as they are characterized by residual ciliary beat frequency [20,34]. To further investigate the diagnostic utility of functional assays, we expanded our study to include pNEC from two individuals with suspected PCD and compound heterozygous biallelic missense variants in *DNAH1*. *DNAH1* is currently known as a PCD candidate gene without sufficient evidence to warrant it being disease-causing (Table 3). Both individuals had a diagnostic workup according to ERS guidelines apart from transmission electron microscopy [6]. HVMA showed near-normal CBF and only slightly reduced beat amplitude and was tested repeatedly (Table 3). In pNEC of both individuals with *DNAH1* variants, DNAH1 protein could not be detected by immunofluorescence staining (Table 3).

In HC pNEC (*n* = 5) CBF was significantly higher at all time points after basolateral treatment with 10 µM clenbuterol compared to untreated control cultures (immediate, 24 h, 48 h; all *p* < 0.0001) (Figure 4a). Clenbuterol treatment had no effect on CBF in *DNAH11* (*n* = 4) or *CCDC40* (*n* = 5) PCD cultures at any time point (Figure 4b,c). In *DNAH1* pNEC (*n* = 2 donors, 2–3 measurements, each with 3–4 filters), treatment with 10 µM clenbuterol led to a significant increase in CBF at all time points (immediately *p* < 0.001; 24 h *p* < 0.01; 48 h *p* < 0.001) (Figure 4d). We additionally measured the mean active area across the culture inserts, and clenbuterol had no significant effect on HC at any time point (18.26–28.53%) (Figure 4e). In PCD pNEC cultures harboring *DNAH11* (0.01–0.20%) and *CCDC40* (0–0.89%) mutations, the mean active area was significantly lower than in healthy controls (Figure 4f,g). Clenbuterol treatment had no effect on the mean active area in cultures harboring *DNAH1* variants (15.25–22.75%) (Figure 4h). The mean active area in cultures from individuals with *DNAH1* variants was comparable to healthy controls (Figure 4e,h).

### 3.3. Effect of Clenbuterol on Mucociliary Transport Velocity in Highly Differentiated Air-Liquid Interface Cell Cultures

Next, we evaluated the effect of clenbuterol on MCTV in ALI cultures of HC individuals, as well as PCD patients with mutations in *DNAH11*, *CCDC40* and individuals with *DNAH1* variants. Acute and chronic treatment with clenbuterol significantly increased MCTV in HC but not in *DNAH11*, *CCDC40* or *DNAH1* cultures (Figure 5a–h, Table A2). MCTV was significantly reduced in *DNAH11*, *CCDC40* and *DNAH1* cultures compared to healthy controls (Figure 5a–h).

## 4. Discussion

In this study, we established and optimized the CRC technology as a robust and scalable platform for the expansion of pNEC in PCD. Our results demonstrate that CRC expansion achieves a high success rate (>90%) and yields sufficient cells for the generation of ALI cultures as well as biobanking, thus overcoming the long-standing limitation of low primary cell counts obtained by nasal brushings. We show that the expanded cells maintain their capacity for differentiation at the ALI, recapitulating in vivo ciliary dysfunction, including abnormal CBF and MCTV. Further, our data supports that this model can be used for the functional assessment of PCD candidate genes and preclinical pharmacological testing, which may improve diagnostics and facilitate precision medicine in PCD.

While cell culture models that mimic the phenotype of the PCD airway epithelium are indispensable for studying disease mechanisms, identifying novel genetic components, and developing therapies, the field has long faced a shortage of convenient models. Obtaining sufficient high-yield material from routine diagnostic brushings remains challenging, and traditional primary cultures have a limited lifespan. Consequently, several in vitro platforms, e.g., induced pluripotent stem cells (iPSC), BMI1-transduced basal cells, and CRC technology, have emerged to address this scarcity of primary tissue, each offering distinct advantages and limitations [35,36,37,38]. iPSCs have become a cornerstone for disease modeling due to their unlimited proliferative capacity and compatibility with CRISPR/Cas9 gene editing [35,38]. iPSC-derived platforms successfully recapitulate coordinated mucociliary flow and genotype-specific defects [36]. While iPSCs successfully capture key features of impaired mucociliary clearance, differences in cellular maturity and architecture mean they may not fully recapitulate all functional aspects of primary epithelial cultures [39]. A notable alternative for long-term modeling is the use of BMI1-transduced basal cells, which bypass replicative senescence to maintain PCD phenotypes for over 19 passages, facilitating extensive deep phenotyping [37]. However, both models require genetic modification and extensive reprogramming timelines.

In line with previous studies using the CRC technology in PCD, we demonstrate that it enhances cell proliferation and lifespan, enabling the generation of large quantities from limited samples while preserving the airway phenotype and disease-specific features, such as ciliary defects, across multiple passages [40,41,42,43]. In addition, we show that the CRC technology is a clinically accessible tool for rapid functional diagnostics and personalized pharmacological testing in PCD from infancy onwards. Unlike traditional primary cultures, which are limited by early senescence, CRC-expanded pNEC can be thawed and re-differentiated, thus providing the capacity for cryopreservation and subsequent biobanking, supporting longitudinal studies [31,41,42,43]. In addition, the CRC technology supports the development of personalized therapies by providing sufficient cell numbers and maintaining disease-relevant phenotypes, which is especially valuable in rare diseases with a heterogeneous genetic background such as PCD [43,44]. Patient-derived primary cell cultures have emerged as the gold standard for the validation of candidate compounds and emerging therapies—such as small molecule drugs, mRNA-based gene replacement, and gene editing in cystic fibrosis—another rare genetic disease [44,45,46].

In our study, we extended functional analyses of CBF to integrated measurements of mucus transport under near-physiological conditions at 37 °C at the ALI. To our knowledge, this is the first study showing significantly reduced MCTV in vitro in a large number of different PCD genotypes. Others have measured MCC at a liquid–liquid interface in nasal epithelial cultures or nasal brushings, e.g., using dry puffball spores [47,48] or fluorescent particles of different sizes in cultures from individuals with *DNAAF6* and *CCDC40* mutations [19,20]. In contrast, we applied fluorescent beads in a minimal liquid volume (0.75 to 2 µL) to the intact mucus layer on top of the highly differentiated pseudostratified epithelial cell layer at the ALI, which may better reflect the physiological mucus layer, mucociliary interactions and mucus transport in vivo.

Clenbuterol treatment induced a significant increase in CBF in cultures from HC and individuals with *DNAH1* variants, whereas no response was observed in *CCDC40* or *DNAH11* cultures. Following clenbuterol treatment, MCTV was significantly increased in HC, but not in cultures harboring *CCDC40*, *DNAH11* and *DNAH1* variants, highlighting the influence of specific genetic variants on drug efficacy. In addition, we provide evidence that CBF alone does not reflect effective directional transport as abnormal ciliary waveform and coordination can persist despite measurable beating. By demonstrating these genotype-specific effects, we provide proof of concept for the utilization of patient-derived CRC ALI cultures for genotype-specific preclinical testing of therapeutic strategies.

Previous studies showed that activation of β2-adrenergic receptors triggers an increase in intracellular cAMP and subsequent activation of PKA, which leads to the phosphorylation of ciliary components [13,14,15,16]. Further, PKA was found in close proximity to the head of radial spoke 3 in mammalian cells [49]. Although we did not investigate the exact mechanism of action, this may explain why the response to β2-adrenergic stimulation is abrogated in severe axonemal abnormalities as seen in CCDC40- or DNAH11-deficient cells. This may also explain the partial response observed in cultures from two patients with *DNAH1* variants. Although DNAH1 as part of the inner dynein arms was missing, we detected other key ciliary components such as DNAH5, RSPH9 and GAS8 respiratory cells of both individuals. As there is very little data on humans, more research is warranted to determine the exact mechanism of action.

*DNAH1* is currently classified as a PCD candidate gene [6]. The most robust evidence for *DNAH1* function comes from male infertility research [50]. *DNAH1* encodes an inner dynein arm heavy chain also expressed in the testis [50]. In affected males, TEM revealed general axonemal disorganization, including mislocalization of microtubule doublets and loss of inner dynein arms [50]. The association between *DNAH1* and PCD was first established by Imtiaz et al., who identified a novel homozygous missense variant in two sisters from a consanguineous Saudi Arabian family [51]. They presented with chronic rhinitis since childhood, frequent coughing and wheezing, nasal discharge, situs inversus and bronchiectasis [51]. Another case study reported four patients with homozygous and compound heterozygous variants in *DNAH1* and the suspicion of PCD. The two patients included in our study presented with normal nNO, only slight abnormalities in HVMA; however DNAH1 was not detectable by immunofluorescence in pNEC. Furthermore, we observed a disconnect in the response to clenbuterol—with an increase in CBF in *DNAH1* cultures without an improvement of mucociliary transport as opposed to healthy controls, which supports the hypothesis that normal CBF alone is not sufficient for proper mucociliary transport. To our knowledge, this is the first report of 2 individuals with compound heterozygous *DNAH1* missense variants, a clinical phenotype consistent with PCD and significantly reduced MCTV in vitro. While this does not warrant a PCD diagnosis according to the current diagnostic guidelines, PCD is suspected in both individuals [6]. Therefore, our data indicate that DNAH1 may be disease-causing.

Despite the high success rate of the CRC technology, several limitations must be acknowledged. First, the limited number of patients and PCD genotypes that were available for this study may limit the generalizability of the data. Second, while the CRC technology facilitates significant expansion, the potential for phenotypic drift across high passages or the selective expansion of specific progenitor subsets cannot be entirely ruled out. Third, as an in vitro model, our system lacks the inflammatory milieu and changes in mucus properties typical for PCD in vivo, which may influence both baseline ciliary transport and the efficacy of therapeutic stimulation in vivo [52]. Finally, although ALI cultures harboring *DNAH1* variants more closely resembled the PCD phenotype in vitro (and in vivo) these findings should be viewed as preliminary and require validation in larger, multi-center cohorts.

## 5. Conclusions

In conclusion, our data support that the CRC technology provides an efficient and scalable platform for expanding patient-derived pNEC that recapitulate in vivo ciliary function and mucociliary transport in patients with PCD. This model may support functional diagnostics, preclinical testing of novel therapies and personalized medicine for PCD and other forms of bronchiectasis.

## Figures and Tables

**Table 1:** Demographics of the study population.

	Healthy Controls	PCD Total	NU	IDA/MTD	ODA/IDA	ODA	PCD Candidates
Number	8	26	10	7	5	4	2
Mean age (years ± SD)	32.93 ± 8.20	29.06 ± 17.16	32.11 ± 18.09	34.37 ± 22.51	28.10 ± 9.63	19.17 ± 11.08	17.45 ± 16.73
Sex (% female)	50	42.86	50	42.86	20	50	50

SD = standard deviation; NU = normal ultrastructure, IDA = inner dynein arm, MTD = microtubular disorganization, ODA = outer dynein arm.

**Table 2:** Genetic background and ultrastructural defect categories of PCD patients.

PCD Cultures	NU	IDA/MTD	ODA/IDA	ODA
*RSPH4A*	1	-	-	-
*DNAH11*	7	-	-	-
*FOXJ1*	1	-	-	-
*HYDIN*	1	-	-	-
*CCDC39*	-	1	-	-
*CCDC40*	-	6	-	-
*DNAAF1*	-	-	1	-
*CFAP300*	-	-	2	-
TTC12	-	-	1	-
*ODAD2*	-	-	-	1
*DNAH5*	-	-	-	1
*DNAI1*	-	-	-	3
Total	10	7	4	5

NU = normal ultrastructure, IDA = inner dynein arm, MTD = microtubular disorganization, ODA = outer dynein arm.

**Table 3:** Clinical characteristics and diagnostic testing of PCD candidates with biallelic *DNAH1* variants.

Donors	PCD Candidate 1	PCD Candidate 2
Sex	f	m
Age at diagnosis	29.28	5.62
Genotype	*DNAH1 c.5140A>T*/*c.11072C>T*	*DNAH1 c.8095A>T*/*c.12767G>T*
Symptoms	Chronic productive cough since childhood, bronchiectasis since childhood	Recurring pneumonias (>5×), chronic productive cough
Situs inversus	No	No
Consanguinity	No	No
Sweat Chloride	Normal	Normal
Antibodies (IgG/IgM/IgA)	IgM low, otherwise normal	Normal
Vaccination antibody titres	Normal	Normal
Respiratory pathogens	Mycobacterium chimaerae Serratia marcescens	Bronchioalveolar lavage negative
Lung function testing ppFEV1 N2-LCI 2.5%	71 13.3	101 6.76
Imaging findings	Bronchial wall thickening, multifocal bronchiectasis, multifocal mucus plugging	Bronchial wall thickening, multifocal mucus plugging
nNO	133 nl/min (ER)	108 nl/min (TB) 123 nl/min (BH)
HVMA		
CBF native CBF post ALI	10.65 Hz 11.02 Hz	8.61 Hz 8.10 Hz
CBP native	Sightly reduced amplitude, hyperkinetic	Near normal
CBP post ALI	Slightly reduced amplitude, hyperkinetic	Slightly reduced amplitude, intercellular coordination reduced
Immunofluorescence staining DNAH1	Missing	Missing
DNAH5	Positive	Positive
GAS8	Positive	Positive
RSPH9	Positive	Positive

Maternal allele, determined by segregation analysis (Trio WES) is shown first. PCD candidate 1 also carries a heterozygous missense variant in *DNAI1* (c.1265T>C). m = male; f = female; age is depicted in years; IgG = immunoglobulin G; IgM = immunoglobulin M; IgA = immunoglobulin A; nNO = nasal nitric oxide; ER = exhalation against resistance; TB = tidal breathing; BH = breath hold; ppFEV1 = percent predicted forced expiratory volume in 1 s; N2 = nitrogen; LCI = Lung clearance index; HVMA = high-speed video-microscopy analysis; CBF = ciliary beat frequency; CBP = ciliary beating pattern.

## Data Availability

The original contributions presented in this study are included in the article/Supplementary Material. Further inquiries can be directed to the corresponding author (M.A.M.).

## Supplementary Materials

The following supporting information can be downloaded at: https://www.mdpi.com/article/10.3390/cells15201871/s1, Video S1.1 HVMA DNAH1_1 side view (50 and 500 fps); Video S1.2 HVMA DNAH1_1 top view (20 and 200 fps); Video S2.1 HVMA DNAH1_2 side view (20 and 200 fps); Video S2.2 HVMA DNAH1_2 top view (20 and 200 fps); Video S3: MCTV_ HC_3; Video S4: MCTV_DNAH11_4; Video S5: MCTV_CCDC40_5; Video S6: MCTV_DNAH1_1.

## Abbreviations

The following abbreviations are used in this manuscript:

ALI	air-liquid interface
BMI1	B lymphoma Mo-MLV insertion region 1 homolog
cAMP	cyclic adenosine monophosphate
CBF	ciliary beat frequency
CBP	ciliary beat pattern
CF	cystic fibrosis
CRC	conditional reprogramming cell
HC	healthy controls
HVMA	high-speed video microscopy analysis
IDA	inner dynein arms
IF	immunofluorescence
iPSC	induced pluripotent stem cells
MCC	mucociliary clearance
MCTV	mucociliary transport velocity
mRNA	messenger ribonucleic acid
MTD	microtubular disorganization
nNO	nasal nitric oxide
NU	normal ultrastructure
ODA	outer dynein arms
PCD	primary ciliary dyskinesia
PKA	protein kinase A
pNEC	primary nasal epithelial cells
ROCK	rho-associated protein kinase
SAVA	sisson-ammons video analysis
TEM	tansmissionelectron microscopy
ZO-1	zonula occludens 1

## Appendix A

### Appendix A.1

Detailed information on all subjects included in the clenbuterol sub study in Table A1.

**Table A1:** Demographics and genotypes of clenbuterol-treated cultures.

Donors	Sex	Age	Gene	Allele 1	Allele 2	ACMG Allele 1	ACMG Allele 2
DNAH1_1	f	29,28	*DNAH1*	c.5140A>T	c.11072C>T	III	III
DNAH1_2	m	5,62	*DNAH1*	c.8095A>T	c.12767G>T	III	III
DNAH11_1	f	21,11	*DNAH11*	c.4333C>T	c.10309T>G	V	III
DNAH11_2	m	33,28	*DNAH11*	c.12363C>G	c.13531_*36del	V	V
DNAH11_3	f	34,11	*DNAH11*	c.4904_4905delAT	c.12899G>A/c.13181C>G	V	IV
DNAH11_4	m	27,89	*DNAH11*	c.12363C>G	c.13531_*36del	V	V
CCDC40_1	f	38,90	*CCDC40*	c.248delC	c.3354C>A	V	IV
CCDC40_2	m	64,52	*CCDC40*	c.248delC	c.248delC	V	V
CCDC40_3	m	3,87	*CCDC40*	c.248delC	c.3129delC	V	V
CCDC40_4	m	20,43	*CCDC40*	c.248delC	c.248delC	V	V
CCDC40_5	f	60,85	*CCDC40*	c.248delC	c.248delC	V	V
HC_1	m	47,44	Healthy Control				
HC_2	f	38,42	Healthy Control				
HC_3	m	31,73	Healthy Control				
HC_4	m	28,65	Healthy Control				
HC_5	f	27,31	Healthy Control				

For individuals with *DNAH1* variants maternal allele is depicted as allele 1 and the paternal allele is depicted as allele 2 as determined by segregation analysis. Subject DNAH1_1 also carries a heterozygous missense variant in *DNAI1* (c.1265T>C). ACMG = American College of Medical Genetics and Genomics; HC = healthy control, m = male, f = female, age is depicted in years.

**Table A2:** Effect of clenbuterol on mucociliary transport velocity.

Group	Control (µm/s) ±SD	Immediate (µm/s) ±SD	24 h (µm/s) ±SD	48 h (µm/s) ±SD
HC (*n* = 5)	1.6	3.5 **	5.1 *	4.9 *
	0.52	1.36	2.54	1.88
*DNAH11* (*n* = 4)	0.15	0.13	0.12	0.13
	0.02	0.01	0.01	0.01
*CCDC40* (*n* = 5)	0.14	0.16	0.15	0.16
	0.02	0.04	0.03	0.02
*DNAH1* (*n* = 2)	0.16	0.20	0.18	0.20
	0.03	0.03	0.03	0.06

HC = healthy control, SD = Standard deviation; * *p* < 0.05; ** *p* < 0.01.

### Appendix A.2

Supplementary videos of the HVMA results of PCD candidate 1 and PCD candidate 2. Videos of MCTV tracings supporting Figure 4a–c.
